# Application of Cyanobacteria as Chassis Cells in Synthetic Biology

**DOI:** 10.3390/microorganisms12071375

**Published:** 2024-07-05

**Authors:** Xueli Liu, Kaixin Tang, Jinlu Hu

**Affiliations:** School of Life Sciences, Northwestern Polytechnical University, Xi’an 710072, China; lxl981012@163.com (X.L.); tangkaixin@mail.nwpu.edu.cn (K.T.)

**Keywords:** cyanobacteria, chassis cells, synthetic biology, biomass production, environmental monitoring

## Abstract

Synthetic biology is an exciting new area of research that combines science and engineering to design and build new biological functions and systems. Predictably, with the development of synthetic biology, more efficient and economical photosynthetic microalgae chassis will be successfully constructed, making it possible to break through laboratory research into large-scale industrial applications. The synthesis of a range of biochemicals has been demonstrated in cyanobacteria; however, low product titers are the biggest barrier to the commercialization of cyanobacterial biotechnology. This review summarizes the applied improvement strategies from the perspectives of cyanobacteria chassis cells and synthetic biology. The harvest advantages of cyanobacterial products and the latest progress in improving production strategies are discussed according to the product status. As cyanobacteria synthetic biology is still in its infancy, apart from the achievements made, the difficulties and challenges in the application and development of cyanobacteria genetic tool kits in biochemical synthesis, environmental monitoring, and remediation were assessed.

## 1. Introduction

Global use of fresh water and fossil fuels (oil, coal, and natural gas) has increased substantially as a result of industrialization and urbanization, resulting in a massive global energy crisis [1]. The extensive use of fossil fuels generates a large amount of greenhouse gases, which retain heat from the sun and are the primary cause of global warming [2]. As one of the most significant primary producers on Earth, cyanobacteria are the only prokaryotes capable of oxygenic photosynthesis. This has made them potentially photosynthetic chassis microbes, sometimes referred to as “green *Escherichia coli*”. Photosynthetic chassis biological cyanobacteria are particularly promising for bioengineering because they collect solar energy through photosynthesis, do not require a biomass carbon source, and contribute to atmospheric carbon dioxide fixation, offering promising strategies for climate change mitigation and carbon neutrality programs. It is noteworthy that one solution to the world’s energy problems in the future could be the capture of solar energy through photosynthesis. Furthermore, cyanobacteria are attractive hosts that thrive in a variety of niches, from oceans to hot springs and deserts, and this geographic diversity gives these hosts an added advantage as well as a reference for future discoveries of excellent cyanobacteria chassis. Due to their greater capacity for photosynthesis and C1 metabolism, cyanobacteria are a promising source of biofuels and a crucial cellular factory to produce other valuable substances. As such, it serves as an excellent microbial chassis and is crucial to both Earth ecology and synthetic biology.

By utilizing design and construction concepts, synthetic biology aims to re-engineer natural systems or create biological systems with desired traits from the bottom up. The potential for developing engineered biological systems is obvious since synthetic biology can make the bioengineering process more predictable by employing standardized biological parts and assembly tools with desirable qualities. Engineered cyanobacteria are highly efficient chassis organisms that have genetically engineered natural cyanobacteria and endowed them with specific metabolic pathways. Currently, engineered cyanobacteria have successfully achieved unnatural functions to produce high-value compounds. This achievement is attributed to the elucidation of the basic biology of photoautotrophic microorganisms, the rapid development of synthetic biology, the improvement of synthetic biology toolbox, and the advancement of genetic modification methods and transformation technologies. This has contributed significantly to the development of cyanobacterial metabolic engineering and significantly broadens the range of functions that can be utilized to design photosynthetic cyanobacteria. As a result, cyanobacteria are rapidly becoming an emerging platform for synthetic biology and metabolic engineering [3]. The most recent research update shows that genetic engineering and synthetic biology of freshwater cyanobacteria are more advanced, and a variety of powerful genetic toolkits are being developed, but more work is still required to modify these species and unlock their full potential for industrial applications [4]. It has been demonstrated in recent years that cyanobacteria can be genetically modified to create both pharmaceutically active chemicals and renewable energy. However, major changes are required to convert this proof-of-concept research into a commercial-scale production process [5].

As people become more conscious of the need to achieve carbon neutrality and combat climate change, they are also becoming more interested in the sustainable manufacturing of valuable substances. In recent years, much emphasis has been placed on biofuels and fine chemicals such as biodiesel, hydrogen, 2, 3-butanediol, ethanol, 3-hydroxypropionic acid, sucrose, and free fatty acids (FFAs), as well as some high-value chemicals such as squalene, alpha-farnesene, limonene, ethylene, resveratrol, p-coumaric acid, caffeic acid, ferulic acid, astaxanthin, lutein, beta-carotene, chlorophyll, phycocyanin, polyunsaturated fatty acids (PUFAs), and pharmaceutical or nutraceutical compounds. In addition, cyanobacteria can be utilized to make a wide range of low-value but highly consumed goods, including polyhydroxyalkanoates (PHAs), polylactic acid (PLA), polyhydroxybutyric acid (PHB), and biodegradable plastic substitutes. As an important microbial chassis, cyanobacteria have attracted much attention due to their ability to use sunlight and CO_2_ to produce high-value chemicals. Cyanobacteria chassis have been modified for many years as “green” shells to generate bulk chemicals, sugars, natural products, biofuels, polymers, and hydrocarbons. However, low product yield and high manufacturing costs make commercial-scale production challenging, even with its numerous remarkable accomplishments [6].

## 2. Optimization Strategy of Cyanobacteria Chassis Cells

In addition to having the functions required for life support, the ideal chassis organism can support the complex gene network that humans have introduced based on their needs. It also has an efficient and stable function and can be used in a variety of fields, including carbon capture, high-value organic material production, chemical and pharmaceutical industries, environmental protection, and scientific experiments [7]. Cyanobacteria have a tiny genome, a straightforward genetic background, faster growth, and easier gene manipulation than eukaryotic algae and plants. As a result, cyanobacteria offer enormous promise as perfect microbial chassis; however, their full potential has yet to be realized [8]. Now, the main challenges to the commercialization of cyanobacteria are the slow growth rate of cyanobacteria chassis organisms, low biomass accumulation rate, and low yield of target products, so there is an urgent need to identify or develop promising strains as the chassis organisms of cyanobacteria biofactory, such as efficient cyanobacteria chassis organisms with rapid growth, strong tolerance, and high biomass accumulation capacity. Here, we summarized the efficient natural cyanobacteria chassis creatures that have so far been found and their related information (Table 1). In addition, we summarize two strategies to screen and improve the engineering cellular robustness of cyanobacteria facing environmental stresses through culture conditions: 1. Adaptive evolutionary strategy, for example, laboratory adaptive evolution. 2. Semicontinuous algal culture supported by machine learning and synthesis.

### 2.1. Develop Cyanobacteria Chassis Organisms with Fast Growth, Strong Tolerance, and High Yield

Cyanobacteria with quick growth and high tolerance have been discovered in recent years, including *Synechococcus elongatus* UTEX 2973, *Synechococcus elongatus* PCC 11801, *Synechococcus elongatus* PCC 11802, and *Synechococcus elongatus* PCC 11901 [9]. Studies have shown that the doubling time of *Synechococcus elongatus* PCC 11802 was 2.8 h under 1000 μmoles photons/m^2^/s light with 1% CO_2_ [10]. Aside from growth benefits, these strains have valuable characteristics such as increased biomass accumulation and resistance to a variety of abiotic conditions. *Synechococcus elongatus* UTEX 2973 is renowned as the fastest-growing cyanobacterium, for which the shortest doubling time was 1.9 h in BG11 medium at 41 °C under continuous 500 μmoles photons/m^2^/s white light with 3% CO_2_, as well as a strain with good resistance to high temperature and intense light conditions [11,12].

It is worth noting that the benefits provided by natural cyanobacteriaceae chassis organisms with natural rapid growth and high tolerance are insufficient to support various external stresses. There has been a lot of basic research published on the development of excellent cyanobacterial chassis properties. Bryant and Ludwig discovered that the marine cyanobacterium *Synechococcus* 7002 can thrive in a wide range of NaCl concentrations [13]. According to Gao et al., the modified cyanobacteria created by Algenol can grow at 50 °C and endure for 16 weeks in conditions containing 1% ethanol [14]. Cui et al. have successfully improved the salt tolerance of the chassis and set the stage for future large-scale seawater cultivation by introducing exogenous transporters and osmotic-compatible substances into *Synechococcus elongatus* UTEX 2973 [15,16]. By enhancing the expression of Group 2 cyanobacteria sigma factor (SigB) coupled to the Type II promoter sequences, Kaczmarzyk et al. recently enhanced the cyanobacteria’s tolerance to both butanol and high temperatures (up to 45–48 °C) [17]. While significant progress has been made, it remains difficult to design one or more genes to produce global variance in strains. Apart from the identification of naturally resilient cyanobacteria chassis, adaptive evolution under laboratory conditions can result in advantageous mutations through heightened screening pressure, like enduring elevated light intensity and salt concentration, as well as high photosynthetic efficiency (explained in the following chapter).

Heterotrophic cyanobacteria are cyanobacteria that grow through heterotrophic metabolism. They do not rely on photosynthesis but obtain energy and carbon sources by intaking external organic matter. Compared with traditional photoautotrophic culture, heterotrophic culture has the advantages of high efficiency, high controllability, and easy industrial production. Jin et al. used a co-cultivation model of heterotrophic and photoautotrophic, and finally achieved the highest biomass concentration level of microalgae culture reported in the world. Furthermore, the advantages of the proposed method in biofuel production were also demonstrated [18]. In general, engineered cyanobacteria as “chassis” are not as productive as heterotrophic cyanobacteria. However, the natural metabolic advantages of heterotrophic cyanobacteria also provide us with a new idea. If effective strategies are developed, higher versions of cyanobacteria chassis may one day replace the green efficient strain.

**Table 1:** Biological characteristics and optimum culture conditions of efficient natural cyanobacteria chassis. Rapid growth was defined as an algal strain doubling time of less than 7 h. High light, salt, carbon, and temperature were taken as references for the environmental conditions.

Species	Features	Cultural Conditions (Optimum)	References
*Synechococcus* sp. PCC 11801	Fast-growing (Doubling time: 2.3 h), Tolerant to high temperature, light intensities, Higher rates of photosynthesis and carbon fixation	38 °C Carbon-free 1000 µmol photons/m^2^/s	[10]
*Synechococcus* sp. PCC 11802	Fast-growing (Doubling time: 2.8 h), Tolerant to high temperature, light and carbon dioxide intensities	38 °C 1% CO_2_ 1000 µmol photons/m^2^/s	[10]
*Synechococcus* sp. PCC 7002	Fast-growing (Doubling time: 2.27 h), Tolerant to high salt light and temperature intensities	38 °C	[19]
*Synechococcus* sp. PCC 11901	Fast-growing (Doubling time: 2 h), Tolerant to high temperature and light intensities, Showed the highest biomass accumulation	Mixed nutrient culture (use the modified AD7 medium)	[20]
*Anabaena* sp. PCC 7120	Heterocystic specialized cells, High nitrogen fixation efficiency	23 °C	[21]
*Synechococcus elongatus* PCC 7942	Fast-growing (Doubling time: 4.9 h), Small genome, Easy to convert, wide salt tolerance	38 °C 300 µmol photons/m^2^/s, Seawater culture	[22]
*Synechocystis* sp. PCC 6803	Fast-growing (Doubling time: 6.6 h), Easy to convert, wide salt tolerance	30 °C 300 µmol photons/m^2^/s	[23]
*Synechococcus* sp. NKBG 15041c	Fast-growing, high glycogen accumulation under nitrogen deficient culture	Seawater culture	[24]
*Synechococcus elongatus* UTEX 2973	Fast-growing (Doubling time: 1.93 h), Tolerant to high temperatures and light intensities, Easy to convert, Showed the highest biomass accumulation	38 °C Freshwater culture	[12]
*Prochlorococcus*	Small genome, Adapted to darkness, Tolerance to high temperature and nitrate	Seawater culture	[25]

### 2.2. Adaptive Evolutionary Strategy

Hundreds of genes are strongly associated with specific habitats. Genes that are differentially abundant in genomes of marine, freshwater, and terrestrial cyanobacteria were found to be involved in light sensing and absorption, chemotaxis, nutrient transporters, responses to osmotic stress, etc., indicating the importance of these genes in the survival and adaptation of organisms in specific habitats [26]. Six et al. studied how light utilization has evolved in the function of temperature and pointed to an important role of temperature in the evolution of the OCP in *Synechococcus* [27]. Cellular physiological phenotypes are often the result of the presence of multiple genetic elements, so it is difficult to effectively modify a single gene or pathway by simple manipulation. Adaptive laboratory evolution (ALE) is a stress-induced approach that improves the tolerance of microalgae under specific environmental stress conditions. Cultivation of host microbes under gradually increasing selective conditions facilitates the accumulation of desired genomic mutations, which finally leads to obtaining required genotypes and phenotypes in stressful environments. ALE has been used to enhance environmental stress tolerance [28], alcohol tolerance [29,30,31], acid tolerance [32], and heavy metal tolerance [33].

Both the low growth rate and the large genome of cyanobacteria are difficulties associated with creating a beneficial mutant by ALE compared with *Escherichia coli* (*E. coli*). In general, compared with *E. coli*, building the evolution of algae strains will need more time. Considering the time-consuming deficiency of the current ALE approaches in cyanobacteria, the introduction of more efficient methods and tools of evolutionary engineering would accelerate the process of identifying and characterizing desired mutants from ALE. A novel method integrating ALE and mutagenesis has also been proposed to improve the efficiency of ALE [28]. To obtain high light- and temperature-tolerant photoautotrophs, Sun et al. constructed the hypermutation system through combinatory perturbations of the genetic fidelity machinery and cultivation environment and increased the mutation rates of cyanobacterium *Synechococcus elongatus* PCC 7942 by three orders of magnitude. Based on the obtained mutants, detailed knowledge about the improved genomic information can be acquired. Overexpression of the shikimate kinase encoding gene in both *Synechococcus* and *Synechocystis* leads to improved high temperature and high light intensity tolerance [28]. Whole-genome sequencing of n-butanol-adapted strains revealed mutations in RpoB and ABC transporters [30]. Among them, RpoB was directly involved in the tolerance to n-butanol, indicating that the amino acid substitution mutation of RpoB enhanced the tolerance of the strain. In addition, ferrous ions can react to release harmful free radicals under oxidative stress caused by n-butanol, while ABC transporters control the intracellular level of ferrous ions to improve strain tolerance. In addition, mutation analysis of the evolved strains revealed that they acquired a wide variety of alcohol resistance abilities due to combinatorial malfunctions of *slr1044* (*mcpA*) and *slr0369* (*envD*), or *slr0322* (*hik43*), and *envD* [31]. Whole-genome resequencing of a robust strain of *Synechocystis* sp. PCC 6803 tolerant to CdSO_4_ identified six genes related to Cd^2+^ tolerance [33].

### 2.3. Semicontinuous Algal Culture Supported by Machine Learning and Synthesis

The photosynthetic biological manufacturing process of cyanobacteria offers a viable technological solution for environmentally friendly development. However, in practical applications, the commercialization of cyanobacteria is limited by economic feasibility and high harvest costs because the photosynthetic productivity and efficiency of cyanobacteria are limited by the light of the day and night cycle, as well as a variety of physical, chemical, and biological environmental pressures and other adverse conditions, and the effective photosynthetic synthesis of a variety of natural or non-natural metabolites is hindered [11]. Semicontinuous algal culture is a kind of culture mode between continuous and batch culture, which can maintain cells at the best growth stage, improve production efficiency, reduce sensitivity to external environmental changes, and reduce production costs [34]. Recent studies have shown that using semicontinuous cultivation of cyanobacteria for the removal of heavy metal ions in water and producing polysaccharides can cause the maximum limit to maintain more than the absorption rate and ensure efficient and sustainable production [35]. Karemore et al. examined the physiological and biochemical responses of Spirulina to various temperature conditions by semicontinuous cultivation in a large-scale outdoor photobioreactor. This cultivation method maximally excluded experimental errors caused by endogenous environmental changes [36].

Thanks to recent developments in artificial intelligence technology, one of the most promising strategies for cyanobacteria chassis cell optimization in synthetic biology is semicontinuous algal growth assisted by machine learning and synthesis. Under the condition of dynamic or unpredictable external, the accurate prediction and complex model processing ability of the model ensure scientific algal culture management. For example, machine analysis and predictive models are used to optimize culture conditions to improve algal growth efficiency and biomass yield. Long et al. developed an aggregation-based sedimentation (ABS) approach in 2022 by altering the fast-growing *Synechococcus elongatus* UTEX 2973 to create limonene, which boosts the surface hydrophobicity of cyanobacteria and improves cell aggregation and precipitation [37]. Furthermore, Lin et al. used a machine learning technique to enhance the light distribution of cyanobacteria cultures and obtained a maximum limonene output of 50 mg/L in *Synechococcus elongatus* UTEX 2973 utilizing a semicontinuous culture [38]. Davis et al. evaluated cultures using a method for maintaining semicontinuous cultures of halophilic microalgal-producing strains, which elucidated the overlapping effects of photosynthesis and osmotic adaptation on biomass accumulation, as well as predicting bioproduct yields under various culture conditions [39]. Machine learning models may find further applications in industrial microbiology and synthetic biology in the future [37]. Future research on semicontinuous algal culture needs to focus on improving efficiency and stability, as well as developing more intelligent and automated control systems. This might include the use of advanced sensors and automation equipment to real-time monitor and adjust the culture conditions, as well as the use of big data analysis and artificial intelligence technology to optimize training strategy. It is important to note that although it has great theoretical potential, this approach still faces challenges in practical applications, including technical difficulty, biological contamination risk, economic feasibility, and large-scale production, which also need to be optimized by researchers in the future.

## 3. Modification of Cyanobacteria Chassis Cells for Synthetic Biology

Synthetic biology creates new networks through chassis cells to meet specific design goals, or it employs chassis biology to engineer certain biological processes, resulting in the creation of new fields or the improvement of current cell functions and behaviors. Because of the basic genetic composition of cyanobacterial cells, their genetic modification will become more viable and reliable. Although cyanobacteria are considered to be a promising chassis model organism adapted to synthetic biology, there are many optimizations in cyanobacteria relative to traditional chassis cells like *E. coli* and yeast, such as growth rate, environmental tolerance, metabolite inhibition, and the addition of more metabolic pathways. In fact, most of the present metabolic engineering and synthetic biology efforts in cyanobacteria are concentrated upon model organisms, such as *Synechocystis* sp. PCC 6803, *Synechococcus elongatus* PCC 7942, and *Synechococcus* sp. PCC 7002, due to the advantages of easy genetic manipulations [40]. Taking this into consideration, the designing and engineering principles to optimize growth performance and enhance metabolic robustness in cyanobacteria should be explored. We summarize four strategies for improving the efficiency of engineered cyanobacteria from both genetic and metabolic dimensions using synthetic biology approaches: 1. Altered endogenous gene expression, for example, inserting heterologous gene cell growth and tolerance-related devices. 2. Introducing heterologous metabolic pathways. 3. Intelligent design of genetic elements. 4. Using artificial intelligence and mathematical models to identify and optimize metabolic stuck points.

### 3.1. Altered Endogenous Gene Expression

Under environmental fluctuations, physiological protective mechanisms will be activated, and the regulatory networks will be shifted in microbial cells to maintain homeostasis and cell survival. Engineering the native stress–response protective systems via genetic manipulations provides another approach to improving the cellular robustness and fitness of cyanobacterial chassis cells. The most used method to modify the cyanobacteria chassis cells for synthetic biology is the modification of the cyanobacteria genome, such as the overexpression or knocking out of endogenous genes. Here, we summarized the recent advances in modifying endogenous genes through genetic engineering in cyanobacteria to enhance cyanobacteria robustness (Table 2). Recently, overexpression of the shikimate kinase encoding gene in both *Synechococcus* and *Synechocystis* led to improved high-light and high-temperature tolerances [28]. Transcriptome analysis indicated that the mutation remodels the photosynthetic chain and metabolism network in *Synechococcus*. Engineering cyanobacteria with resistance to environmental stresses is of great economic significance for promoting scaled cyanobacteria cultivations. Overexpression of *hspA* and *osmotin* in *Synechococcus elongatus* cells confers multiple tolerances (high temperature, high light intensity, and salt tolerances) under outdoor cultivation [41]. Cyanobacteria have been successfully demonstrated to produce many value-added bioproducts, such as ethanol, hexane, and butanol [42].

### 3.2. Introducing Heterologous Metabolic Pathways

The introduction of the heterogenous pathway for synthesizing products can alter the whole cellular metabolism. Successful implementation of synthetic metabolic pathways in microbial hosts for bioproduction requires the expression of heterologous genes, typically by cloning them into plasmids or integrating them into chromosomes. Heterologous expression in cyanobacteria largely relies on gene integration, either into the chromosomes or endogenous plasmids. This technique is a promising strategy for both the transformation of the cyanobacteria chassis autonomous metabolic system and the improvement of product titer. Dexter et al. heterologous expression of *Sphingopyxis* sp. USTB-05 microcystinase (MlrA) in *Synechocystis* sp. PCC 6803, compared with the natural MIrA host (*Sphingomonas* sp. ACM 3962), the activity of the synthesized biocatalyst against microcystin-LR was increased by about three times [51].

There are also cases of heterologous expression of biosynthetic gene clusters isolated from complex genomic samples in cyanobacteria. Taton et al. created an *Anabaena* strain containing *Staphylococcus elongatus* homologous sequences to facilitate the transfer of biosynthetic gene clusters in *Synechococcus elongatus* PCC 7942 and *Anabaena* PCC 7120 [52]. The two model strains successfully produced JHB by expressing a 28.7 kb cryptomaldamide biosynthetic gene cluster from the Marine cyanobacterium *Moorea*. In addition, the expression of heterologous fusion proteins in cyanobacteria also provides us with a promising idea. Heterologous protein expression is the process of expressing a target gene in a non-native species so that researchers can better understand the structure and function of the protein. Currently, cyanobacteria have been widely used in the expression of heterologous proteins. For example, phycocyanin in cyanobacteria has been used as a vector for fusion constructs to express heterologous proteins from plants, bacteria, and humans. Recently, there have been studies on the fusion expression of the interesting heterologous protein “P” with the abundant CpcB β-subunit of phycocyanin (PC). The fusion protein accumulates in a soluble and stable form in the cytoplasm of cyanobacteria in a certain way, successfully improving the photochemical charge separation rate and electron transfer activity of the PSII photosystem while retaining the activity of the heterologous “P” protein. Plant-derived isoprene synthetase, β-camylene synthetase, geranyl diphosphate synthetase, and geranyl linalol synthetase were also shown to be overexpressed while preserving catalytic function [53].

### 3.3. Artificial Intelligence Design Genetic Elements

As a green chassis to capture CO_2_ for biotechnological applications, the genetic toolbox for cyanobacteria is still a limited factor. Nowadays, the focus in metabolic engineering research is shifting from massive overexpression and inactivation of genes towards the model-based fine-tuning of gene expression. The acquisition of well-characterized biological parts can promote diverse and targeted control of gene expression. The judicious choice of promoter to drive gene expression remains one of the most important considerations for synthetic biology applications [54]. Constitutive promoter sequences isolated from nature are often used in laboratory settings or small-scale commercial production streams, but unconventional microbial chassis for new synthetic biology applications require well-characterized, robust, and orthogonal promoters. Computer-aided design combined promoters can find out the promoter sequence to promote gene expression through simulation and calculation, which provides more elements for synthetic biology and helps researchers study more complex biological systems. Seo et al. provided a deep-learning-based generic framework to design and evaluate synthetic promoters for cyanobacteria using generative models, which were in turn validated with cell-free transcription assay [55]. This approach, combining in silico and in vitro studies, will provide a foundation for the rapid design and validation of synthetic promoters, especially for nonmodel organisms. A research team from the Chinese Academy of Sciences has developed the ProEnsemble machine learning framework, which combines the advantages of automation and machine learning to improve the speed and efficiency of chassis development, shorten the development cycle, and reduce costs [56]. The Tsinghua University research team proposed DeepSEED, which is a kind of expert knowledge with big data integration, to study the synthesis of the promoter artificial-intelligence-aided design method [57]. Using this method, successfully obtaining a batch sequence diversity is strong, and the functioning is better than the natural sequence of the new promoter. In the future, with the development of artificial intelligence technology, this method is expected to predict and design promoters more accurately through deep learning and big data analysis further improve the efficiency and specificity of gene expression.

### 3.4. Using Artificial Intelligence and Mathematical Models to Identify and Optimize Metabolic Stuck Point

**Figure 1:**
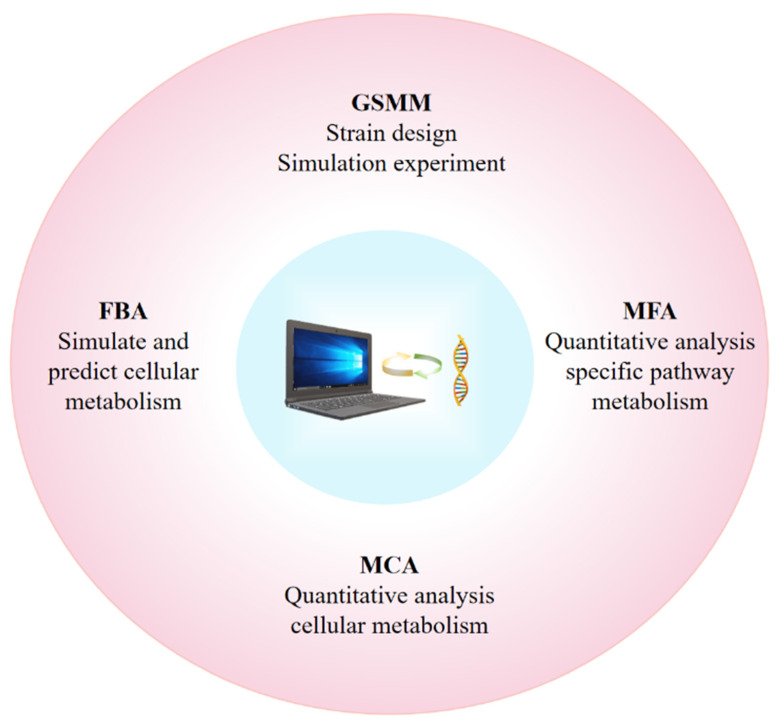
Mathematical models of commonly used metabolic engineering and their applications. The pink circle listed in the figure is four common metabolic model, which can identify metabolic card points from different angles and degrees.

In the field of synthetic biology, metabolic control and optimization are key research directions. A metabolic process is the process of some chemical reactions and substance transformation in organisms. Its control and optimization can improve production efficiency and yield and reduce production costs. However, parameter estimation is a crucial step in metabolic control and optimization. In recent years, the use of artificial intelligence technology to optimize research models for metabolic engineering has become a hot research area, and it has shown significant results in practice. Therefore, computer simulation can provide a systematic approach to developing ideal metabolic networks that can theoretically maximize production while avoiding errors. At present, most of the cyanobacteria production pathway engineering studies not only rely on experience but also use artificial intelligence and mathematical models to predict and identify some metabolic plug-points in advance, systematically analyze and understand the primary metabolism and regulation of cyanobacteria, and improve the yield of target products, which is crucial for the application of cyanobacteria in synthetic biology. With the development of synthetic biology, researchers are now incorporating rich omics data into metabolic models to increase the predictive capacity of computational models and their accuracy in handling perturbed conditions. These traditional metabolic models have helped to resolve some biotechnology-related issues [58]. When cyanobacteria cultures reach optimal cell density, light and carbon are insufficient to nourish all cells. Metabolic models can be designed to achieve the maximum yield of the target product. For example, accumulating biomass and metabolites during the growing period and increasing the carbon allocation of the product. These applications also have been used to uncover the potential metabolic capacity of cyanobacteria, investigate the natural regulatory pathways of cyanobacteria metabolism, and design strategies to achieve desired outcomes [59]. However, the choice of different flow analysis techniques also has a great impact on the study of cyanobacterial metabolism and control [60]. Here, we summarized four commonly used mathematical models for metabolic engineering (Figure 1).

Determining the rate-limiting processes is the primary difficulty for cell factories based on cyanobacteria chassis. Traditional trial-and-error methods for genetic target identification are time-consuming and provide little useful information. At present, researchers have constructed thousands of metabolic models of species, which are widely used in biological cell factory design. Genome-scale metabolic network models (GSMM) is a systems biology tool that describes the relationships between genes, proteins, and processes. It is a mathematical model that systematically describes cell metabolism and completes experiments through computer modeling instead of time-consuming and laborious or unachievable pre-experiments, which improves research efficiency. It is also useful for studying the steady-state and dynamic behavior of metabolic networks, as well as identifying rate-limiting steps in metabolic pathways [61]. GSMM has been used to analyze the intracellular metabolic regulation of industrial microorganisms, especially to screen metabolic engineering targets and improve the yield of target products [62]. The limitation of the current GSMM is the lack of physiological information constraints. Therefore, researchers have long been committed to integrating various types of information in GSMM to improve models. The enzyme-constrained model (EcModel), compared with the classic GSMM, has better prediction performance and wider application prospects [63]. However, on the one hand, integrase constraint requires many enzyme parameters, and the lack of parameters hinders the popularization of enzyme constraint models. On the other hand, there is a high threshold for the construction and use of enzyme constraint models, which is not conducive to the promotion and application of models.

The Flux Balance Analysis (FBA) model in metabolic engineering is a mathematical tool used to simulate and optimize the behavior of metabolic networks within cells. By building a large-scale model of the metabolic network, FBA can predict the flow distribution of various metabolites within the cell under certain conditions and how these flows affect cell growth and product formation [64]. It is possible to fully characterize the light response of photosynthesis, highlighting the variations in metabolism under various environmental circumstances. Nevertheless, the drawback of FBA is that all cyanobacteria must alternate their diurnal metabolism during the diurnal cycle, and a pseudohomeostasis must be established [65].

Metabolic flux analysis (MFA) provides insight into the state of cellular metabolism under specific conditions by determining the production and consumption rates of metabolites in biological systems. It can be used to rationally increase the yield of desired products, rigorously identify bottleneck stages that limit product creation, and compare wild types with design strains [66]. The analysis of ^13^C metabolic flux (^13^C-MFA) has emerged as a modeling technique for measuring intracellular flux. A few researchers have designed strains using ^13^C-MFA; for example, *Synechocystis* sp. PCC 6803 was designed to generate ethylene. With technological advancements, isotope nonfixed metabolic flux analysis (INST-MFA) has allowed for more detailed information on inactive pools, reversible fluxes, possible metabolite pathways, and metabolic bottlenecks than FBA, which can only provide net fluxes. Cheah et al. determined the metabolic bottleneck preventing *Synechococcus elongatus* PCC 7942 from producing isobutyraldehyde by utilizing the INST-MFA approach and the metabolic profile. Downregulating the process between phosphoenolpyruvate carboxylase and pyruvate dehydrogenase resulted in a higher production of aldehydes [67].

Metabolic Control Analysis (MCA), a mathematical model in metabolic engineering, is a quantitative analysis method and theoretical framework for studying metabolic regulation in cells. It is used for reactions with high flux control coefficients and can offer direction for rate-limiting steps. Currently, MCA has been shown to boost the synthesis of cyanobacterial products such as lactic acid [68], limonene [69] and ethanol [70]. Sengupta et al. discovered in 2020 that *BiBPase* becomes the critical node of the succinate production pathway that limits rate; overexpression of the *BiBPase* gene eventually removes this rate-limiting step [71].

To increase the yield of target products, cyanobacteria-related genetic information has been predicted using a number of algorithms, including OptGene, minimization of metabolic adjustment (MOMA), OptKnock, and OptForce [72]. With its fast-processing speed and capacity to optimize nonlinear goals, OptGene can theoretically produce effective solutions and accomplish the intended goal [73]. OptKnock is an optimized metabolic engineering algorithm that optimizes the production of biosynthetic products by identifying the best gene knockout strategy. It through mathematical optimization to identify knock out some of the genes in metabolic network achieve maximize product flux [74]. Using the OptForce design algorithm, Lin et al. overexpressed genes involved in the pentose phosphate pathway and geranyl pyrophosphate synthase to maximize the efficiency of limonene production [38]. MOMO was used to compare differences between two metabolic states and to minimize metabolic adjustment. It is commonly used to analyze the metabolic differences between wild-type and mutant strains to resolve the effect of gene knockout or icon on cell metabolism [75].

Despite successful cases, the study of metabolic process control and optimization still faces many challenges, for example, how to better integrate experimental data with model predictions, how to further improve the efficiency and accuracy of the optimization algorithm, etc. Future research can focus on the development of more advanced algorithms and the use of big data and machine learning techniques to further improve the prediction ability and optimization effect of models.

## 4. “Green Engineering” Built in the Chassis Cells of Cyanobacteria

In recent years, with the rapid development of synthetic biology and gene editing technology, the research and application of cyanobacteria cell factories have made remarkable progress. Although the ability of engineered cyanobacteria to synthesize natural or unnatural metabolites can be greatly improved under laboratory conditions, their large-scale application is technically and economically limited. For example, first, it is difficult to maintain stable synthesis of target products in the face of external environment fluctuations. Second, under industrial-scale cultivation, cyanobacterial cell biomass harvesting and target product extraction are extremely energy-intensive and complex processes, which greatly reduce the cost competitiveness of the whole technology chain. However, it is important to note that in the commercial application of such products, ease of harvesting is critical. Compared with heterotrophic chassis, the production concentration and productivity of cyanobacteria are relatively low and the harvest of products is particularly difficult. In particular, the secretion of the resulting chemicals into the culture medium can limit the commercialization process. In addition, the state of the production material determines the harvesting method and its difficulty, so it is particularly important to study the harvesting method of products in different states. Because the generated cell inclusion body particles are easy to harvest, the production of polymer products is easier to harvest than other state products. Cyanobacteria are ideal cell factories for generating low molecular weight compounds with water-soluble biological products. Tan et al. observed that cyanobacterial synthesis of water-soluble compounds is often minimal, which is a key impediment to the commercialization of cyanobacteria cell factories [76]. When water-soluble compounds are existent in extremely low quantities, collection and purification are time-consuming and labor-intensive processes. Because medium- to long-chain hydrocarbons are often insoluble in water, they are simpler to acquire by centrifugation than soluble low-molecular-weight compounds. Furthermore, gaseous substances are easier to separate than liquid ones, requiring less time and money.

As a result, we categorize the adapted production substances into polymer substances, water-insoluble liquid substances, and gases and summarize the harvest methods and advantages of the three states of products and recent progress in increasing yields [7,9,11]. We proposed some feasible harvesting methods for a few high-value compounds that are soluble in water (Table 3).

### 4.1. Macromolecular Substance

Cyanobacteria, as a natural host for efficient carbon resource conversion, have gained increasing interest in the field of polymer synthesis [99]. Polymer materials are more valuable and have a greater range of applications. Additionally, there are more benefits in solving the cyanobacteria cell factory harvest problem as compared with low-molecular-weight compounds, since the cyanobacteria cells only make up a small portion of the culture medium’s volume, and because the flocculation treatment and polymer biosynthesis can be integrated flawlessly. Cationic charges have been widely used to increase the adsorption and flocculation dispersion of polymers [100]. Given its simplicity of harvest, we believe it could be a viable industrial strategy for future cyanobacteria cell factories.

#### 4.1.1. PHA

PHA is a biodegradable plastic produced by cyanobacteria cell factories and the most promising “green plastic” in terms of biocompatibility and biodegradability. The biopolymer polyhydroxyalkanoate (PHA) can be synthesized by *Synechocystis* sp. PCC 6803 [101]. According to the study, PHA synthase activity in *Synechocystis* sp. PCC 6803 increased by twofold upon the introduction of PHA biosynthetic operons from *Ralstonia eutropha*. 3-hydroxybutyrate (3-HB) is a prerequisite for PHA biosynthesis. Wang et al. designed *Synechocystis* sp. PCC 6803 (overexpressing thioesterase and acetyl-CoA reductase and knocking out polyhydroxy butyrate polymerase) to produce 3HB up to 533 mg/L [77].

#### 4.1.2. PHB

The most common form of PHA, polyhydroxybutyrate (PHB), accumulates naturally in *Synechocystis* sp. PCC 6803 under conditions of nitrogen or phosphorus deficiency (particles up to 0.8 microns, 27 mg/L) and can replace the widely used petroleum-derived plastic polypropylene (PP). It also has potential uses in the pharmaceutical and medical industries [99]. The study discovered that large amounts of PHB accumulation can result from disruption of the genes *sll0461*, which encodes gamma-glutamyl phosphate reductase (proA), and *sll0565*, which encodes an unknown protein [102]. Transposon insertion is also utilized to investigate the PHB biosynthesis pathway. It was recently discovered that by constructing an engineered strain expressing the *phaCAB* allogeneic gene, PHB production could reach 420 mg/L (16.7% of DCW), with the greatest specific yield of 75.2 mg/L/d [78].

### 4.2. Water-Insoluble Liquid Substance

Cyanobacteria may use carbon dioxide to convert into water-insoluble liquid products, such as medium–long-chain hydrocarbons. Hydrocarbons are ideal fuel ingredients to use as raw materials to produce rubber, plastics, fibers, solvents, and industrial chemicals. Since hydrocarbons are extremely hydrophobic and volatile, they separate on their own when the aqueous phase of cells and media spreads. They accumulate above the liquid culture in the gas phase of a completely enclosed reactor. Therefore, the harvesting problem of hydrocarbons presents three kinds of advantages: (1) the natural secretion of cells; (2) it is easy to separate from the water medium; (3) it can be used without further processing. Thus, a sustainable and promising approach to overcoming challenges with product harvesting is the production of medium- and long-chain hydrocarbons using direct carbon capture.

#### 4.2.1. Limonene

Within the terpenoid compound class, limonene is a valuable natural substance with potential commercial applications. It is often utilized as a medicinal component, fuel, fragrance, and other applications. Citric acid synthase was found to be heterogeneously expressed in cyanobacteria, and the bacteria were able to successfully generate limonene. Lin et al. engineered *Synechococcus elongatus* UTEX 2973 to produce a commercially available terpene synthetase crtE, resulting in a 2.5-fold increase in limonene production (16.4 mg/L, 8.2 mg/L/d), which is eight times higher than previously reported limonene production in other cyanobacterial species [82]. Shinde et al. improved *Synechococcus elongatus* PCC 7942 by overexpressing primary sigma factors, which led to a maximum production of 4.3 mg/L/d and a titer of 19 mg/L after seven days of limonene. Therefore, enhancing photosynthesis and substrate input is an important strategy to improve the productivity of secondary metabolic pathways [103].

#### 4.2.2. Biodiesel

Energy-storing substances, including diacylglycerol (DAG), triacylglycerol (TAG), and starch, are abundant in cyanobacteria and algae. These chemicals may be collected and utilized to make biodiesel. However, a significant barrier to the commercial manufacturing of biodiesel is the significant amount of glycerol that is produced as a byproduct of the lipid extraction process [99,104]. According to Wang et al., preventing the synthesis of starch may be a useful strategy for boosting the synthesis of lipids, which might lead to an increase in the generation of biodiesel [104]. Furthermore, physical cell breaking and subsequent chemical solvent extraction are necessary for the conventional downstream recovery of lipids, which comes at a significant financial expense. Liu et al. developed a more economical green recovery technique in *Synechocystis* sp. PCC 6803 by employing CO_2_ limitation to stimulate promotor to regulate lipolysis, which breaks down membrane lipids into free fatty acids (FFA), in order to initiate cell lysis and ease lipid extraction during harvest [105]. Cyanobacteria biodiesel has significant advantages in terms of environmental performance, pollution reduction, renewability, and safety, but it has disadvantages in terms of energy density, storage problems, and feedstock constraints.

#### 4.2.3. FFAs

Free fatty acids (FFAs) are a class of industrial compounds frequently utilized in biofuels. Numerous genetic engineering techniques have been used to increase the synthesis of FFA in cyanobacteria. By removing fatty acid activation gene *aas* (slr1609) and overexpressing acetyl-CoA carboxylase (ACC) to push metabolic flow to FFAs, Liu et al. degraded and inactivated FFAs. Lastly, the engineered cyanobacteria strain has a maximum FFA secretion capacity of 197 mg/L [106]. Recently, Eungrasamee et al. significantly increased the production of free fatty acids (238.1 mg/L) by mutating the *aas* and *sll1951* genes of *Synechocystis* sp. PCC 6803, especially under nitrogen deficiency conditions [107]. The increased FFA production of engineered cyanobacteria is mainly related to thioesterase (*tesA*) and its related genes, and according to the study of Eungrasamee et al., it is speculated that genes in the glycogen synthesis pathway are also related to FFA expression [106,107]. It also shows that the deletion of glycogen synthesis key gene *glgC* could significantly increase the intracellular total fatty acid content. Recent studies have also shown that the introduction of exogenous free fatty acids into cyanobacteria can guide the activation of intracellular fatty-acid-containing biomolecular pathways, which provides a new idea for the synthesis of free fatty acids in cyanobacteria [108].

### 4.3. Gas

Gas products are insoluble in water and can be easily harvested without any further process engineering. The kind, nature, and actual state of the gas all affect the gas flux measurement method. Du et al. investigate the interaction impacts of different growing seasons and greenhouse management strategies on agricultural greenhouse gas emissions by using a gas chromatograph equipped with a flame ionization detector and an electron capture detector for concentration analysis of CO_2_ and N_2_O, respectively [109]. Due to the easy collection and measurement of gas products, it also becomes a feasible suggestion for future research topics.

#### 4.3.1. Ethylene

Ethylene is a volatile gas product used as a raw material for petroleum. The autotrophic synthesis of ethylene from cyanobacteria was successfully achieved by genetically engineering cyanobacteria to express the ethylene-forming enzyme (*efe*) gene from *Pseudomonas syringae*. Carbonell et al. found the overexpression of the key enzyme gene *efe* can improve the efficiency of ethylene production [110]. Claudia et al. heterogenetically expressed highly active phosphoenolpyruvate carboxylase in the central carbon metabolic pathway of cyanobacteria, which enhanced the carbon supply of the tricarboxylic acid (TCA) cycle and increased ethylene production [111]. In addition, in the regulation of cyanobacteria metabolism, NtcA is an important global transcriptional regulatory factor in the process of primary carbon and nitrogen metabolism. NtcA exerts a negative regulatory effect on the promoter PcpcB that controls *efe* expression. Huilin et al. obtained recombinant strains by partially missing NtcA and four copies of *efe*. The ethylene production rate was increased to (2463 ± 219) μL/(L/h/OD_730_) [86].

#### 4.3.2. Hydrogen

Hydrogen is a carbon-free, high-energy cyanobacteria fuel. It is one of the most promising clean fuels, and cyanobacteria and algae naturally produce hydrogen as a secondary metabolite to balance energy. The key role of cyanobacteria in the photosynthetic production of hydrogen is hydrogenase, which catalyzes the reduction of protons to hydrogen [112,113]. It has been found that photosynthetic hydrogen production can be achieved by the fusion of hydrogenase and photosystem in vivo. It is important to note that this process is not 100 percent efficient, as cyanobacteria also produce oxygen and other by-products during photosynthesis. However, to optimize this process, Kruse et al. increased the electron flux of hydrogen biosynthesis catalyzed by hydrogenase and blocked the cyclic electron transfer around PSI. The results show that the H_2_ precipitation rate is increased by more than five times under certain conditions [114]. As mentioned in Antal and Lindblad, S starvation increased the H_2_ production of *Synechocystis* sp. PCC 6803 by more than fourfold. Stimulation with S starvation, CH_4_, and low-extracellular pH could enhance the H_2_ production of *Synechocystis* sp. PCC 6803 during fermentation; therefore, stimulation of H_2_ production in the presence of CH_4_ opens new possibilities for increasing H_2_ production [115]. In addition to photosynthesis, hydrogen production by engineered cyanobacteria can also be obtained by glycogen fermentation. Some studies have shown that the hydrogen yield of endogenous glycogen fermentation in cyanobacteria exceeded 90% of the theoretical maximum (3.8 mol H_2_/mol glucose) [116]. After that, a two-stage recycling system for hydrogen production by photosynthetic culture and glycogen fermentation was established, which exceeded that of ordinary glycogen fermentation in terms of yield and efficiency [117]. In the future, the combination of culture methods and endogenous genetic modification can be considered as a promising idea to improve the efficiency of hydrogen production.

### 4.4. Water-Soluble but High-Value Products

Cyanobacteria have enormous potential for being engineered to overproduce massive quantities of valuable products [118]. Natural compounds such as terpenes and phenyl-c products have been used in the manufacture of cyanobacteria. Ni et al. constructed a photoautotrophic synthesis platform of *Synechococcus elongatus* PCC 7942 that can synthesize resveratrol, p-coumaric acid, caffeic acid, and ferulic acid [119]. However, in the face of water-soluble products, how to efficiently harvest is still a problem to be solved. For some water-soluble plant-derived natural products, cyanobacteria have better adaptability, easier access to reducing energy, lower dependence on expensive substrates, and easier harvest than other water-soluble products expressed exotically, which can be further converted into some other precious and valuable natural products [120]. Therefore, it is also one of the promising strategies for efficient harvesting of water-soluble products. We also provide workable recommendations for strain selection and culture in addition to product selection. Since single-cell cyanobacteria are only 0.3–0.5 μm in size, choosing filamentous strains with larger sizes (200 μm), like *Anabaena* [121], *Nostoc* [122], and *spirulina* [123], can make the products more easily separated from the growth medium through filtration and eventually naturally form cell aggregates. It is one of the feasible strategies for efficient product recovery. Furthermore, modifying the morphology of *Synechococcus elongatus* PCC 7942 has been demonstrated to effectively augment cell size, consequently amplifying precipitation and elevating biological yield [124]. Strain selection can be enhanced ecologically by employing multi- and mixed-culture techniques to boost productivity and stability. Studies have also demonstrated the potential benefits of high-yield production using farming techniques that use nutrients from wastewater [125]. Furthermore, the size of the photobioreactor plays a vital role in controlling cell growth and chemical output. Most of the cyanobacteria production studies reported to date have been conducted under small-scale conditions. In contrast, growing engineered cyanobacteria strains in photobioreactors with high-intensity light and high CO_2_ concentrations may considerably boost product titers [95].

#### 4.4.1. Ethanol

Ethanol was the first biofuel product to achieve commercial application. The biosynthetic pathway of ethanol in cyanobacteria is usually through the expression of foreign substances or through the regulation of naturally regulated pathways in algae. In 1999, it was reported that *Synechococcus elongatus* PCC 7942 had been genetically engineered to produce up to 230 mg/L of ethanol. In 2009, Dexter and Fu integrated pdc/adh expression boxes onto PpsbA2, a photoinduced strong promoter on the chromosome of *Synechocystis* sp. PCC 6803, and engineered cyanobacteria to produce approximately 550 mg/L ethanol under intense light (1000 μE/m^2^/s) [126]. Recently, Gao et al. expressed pyruvate decarboxylase in *Synechocystis* sp. PCC 6803 and optimized the promoter strength. At the same time, the overexpression of endogenous ethanol dehydrogenase successfully increased the ethanol yield from 0.5 g/L to 5.5 g/L [127].

#### 4.4.2. Sucrose

Sucrose is a representative of compatible substances and one of the main raw materials for synthetic biofuels. Sucrose secretion from cyanobacteria cells was first reported by Ducat et al., who introduced the *CscB* gene from *E. coli* into *Synechococcus elongatus* PCC 7942 [128]. Traditional cyanobacteria can achieve high sucrose synthesis under salt stress, which is considered an ideal carbon source to support heterotrophic microorganisms. *Synechococcus elongatus* UTEX 2973 produced 8 g/L sucrose under salt stress culture [129]. On this basis, after the introduction of *CscB* gene, the cell sucrose production of this strain exceeded 94% [90].

#### 4.4.3. Lysine

Lysine is an essential amino acid that can be used as an additive in food, feed, cosmetics, and pharmaceuticals, and in recent years, it has also been applied to the photoautotrophic production of cyanobacteria cells. Recently, a study showed that engineered lysine overproduction (556.3 ± 62.3 mg/L) by introducing feedback-resistant copies of aspartic kinase (encoded by *lyscbr*) and lysine export protein (encoded by *ybjE*) from *E. coli* to *Synechococcus elongatus* UTEX 2973. It also exceeds the previously reported bioproduction of photoautotrophic lysine [92].

#### 4.4.4. Succinic Acid

Succinic acid is an important platform used in medicine and agriculture. In the traditional sense, the genes encoding α-ketoglutarate decarboxylase and succinic semialdehyde dehydrogenase were expressed into cyanobacteria, and succinic-acid-producing cyanobacteria strains were obtained [130]. Sengupta et al. overexpressed sedoheptulose-1 and 7-diphosphatase and knocked out the glycogen synthase A (*glgA*) gene and the succinic dehydrogenase B subunit (*sdhB*) gene, resulting in the host strain *Synechococcus* sp. PCC 11801 producing 0.93 g/L succinic acid (within five days), which is more than two times higher than the best titer that has been reported [71].

## 5. Application of Modified Cyanobacteria Chassis Cells in Environmental Monitoring and Remediation

Environmental pollution is a process that causes adverse effects on the function and value of the environment due to the excess of substances or energy in the environment caused by human activities or natural phenomena [131]. Environmental pollution is one of the critical issues facing the world today, as various pollutants accumulate in the environment, posing serious threats to human health and ecological balance. To effectively monitor and remediate environmental pollution, new technologies and methods are needed to achieve rapid, accurate, cheap, and continuous detection and removal of pollutants in the environment. Cyanobacteria are a group of photosynthetic prokaryotes that have various special physiological functions and metabolic abilities. By using their photosynthesis and metabolic engineering, they can transform organic and inorganic substances in the environment into harmless or useful substances and have very wide applications in environmental monitoring and remediation [132]. Natural algae’s environmental detection and repair ability is mainly manifested in environmental monitoring, water purification, ecological restoration, etc. Cyanobacteria are sensitive to environmental changes in water quality, and their growth status can be used as an indicator of water pollution. In addition to providing an oxygen supply to other organisms through photosynthesis, certain cyanobacteria are converted to plant-usable nitrogen compounds through nitrogen fixation, providing rich nutrients to soil and other plants. However, natural cyanobacteria are limited in their ability to monitor and remediate the environment and need to be modified and optimized by synthetic biology techniques. By using synthetic biology techniques, cyanobacterial chassis cells can be precisely designed and modified, enhancing their function and performance in the environment and expanding their application scope and potential in environmental monitoring and remediation [133]. This section will introduce the application of modified cyanobacterial chassis cells in environmental monitoring and remediation and look forward to the future development direction and possible challenges.

### 5.1. Application of Modified Cyanobacteria in Environmental Monitoring

Environmental monitoring is the process of using various technologies and methods to observe, analyze, and evaluate the distribution, change, and impact of substances or energy in the environment [134]. Biosensors can use biological organisms or their components to produce measurable response signals to specific substances or conditions, which can achieve rapid, accurate, cheap, and continuous monitoring of harmful or beneficial factors in the environment [135]. Biosensors usually consist of a sensing element and a reporting element. The sensing element is a gene or gene combination that can respond to the input signal. The reporting element is a gene or gene combination that can produce an observable output, such as fluorescent protein or bioluminescent protein. Biosensors use the interaction between biomolecules or cells and pollutants to produce measurable signals, which can reflect the type and concentration of pollutants [136]. Cyanobacterial biosensors are a type of genetically encoded sensors that use the luminescence [137], fluorescence [138], or pigment [139] characteristics of cyanobacteria to produce signals, which can detect different environmental factors within cells [140]. Cyanobacterial biosensors are mainly used for detecting heavy metals, pesticides, and other organic and inorganic pollutants in water bodies. Here are some application cases of constructing biosensors using modified cyanobacterial chassis cells.

Heavy metal pollution is a common water environmental problem, which has adverse effects on the ecosystem and human health [141]. Peca et al. [142] constructed a bioluminescent cyanobacterial biosensor for detecting the presence and concentration of nickel ions1. This method used the promoter of the nickel transporter gene in cyanobacteria, which is related to the bioluminescent protein gene, forming a regulatable luminescence system with high sensitivity and specificity. Wong et al. [143] evaluated the performance of a cyanobacterial whole-cell fluorescent biosensor for detecting heavy metal and pesticide pollution. This method used the expression of the fluorescent protein gene in cyanobacteria as a biomarker for pollutants, reflecting the type and level of pollutants by the change in fluorescence intensity, with the advantages of being fast and simple.

Due to the widespread use of chemicals in industry and agriculture, many potential organic pollutants are released into the environment. These organic pollutants have carcinogenic and mutagenic properties, and their wide distribution poses a serious threat to human health and the loss of ecological diversity [144]. Shao et al. [145] developed a novel cyanobacterial biosensor for detecting the concentration of the herbicide atrazine. This method used the composition and function of the photosynthetic electron transport chain in cyanobacteria, which is related to an electrode, forming an electrochemical biosensor, reflecting the inhibitory effect of the herbicide by the change in current with the characteristics of high sensitivity, good stability, and strong repeatability.

Compared with other biosensors, constructing biosensors using modified cyanobacterial chassis cells has the following advantages: 1. High sensitivity and resolution: Cyanobacterial biosensors can monitor subtle signal changes in real time within cells, detect low concentrations of pollutants, and distinguish different types and levels of pollutants; 2. High dynamic range: Cyanobacterial biosensors can adapt to different signal intensities, detect pollutants in a wide concentration range, and regulate their own response intensity, avoiding signal saturation or attenuation; 3. High stability and low cost: Cyanobacterial biosensors can be used for a long time under different conditions, are not easily affected by environmental factors or lose activity, and can use light energy and inorganic substances in water for self-sufficient growth, without the need for additional energy or nutrients, reducing the cost and maintenance difficulty of biosensors [146].

### 5.2. Application of Modified Cyanobacteria in Environmental Remediation

Environmental remediation is the process of using various technologies and methods to purify and restore an environment that has been polluted [147]. Biodegradation is one of the main methods of environmental remediation [148], which is the process of using biological organisms or their components to metabolize or transform pollutants into harmless or useful substances. By using genetic engineering and omics technologies to modify the metabolic pathways and functional expression of microorganisms, their ability and efficiency to degrade pollutants can be improved, thus achieving renewable, low-carbon, and efficient environmental remediation [149]. Cyanobacteria are a group of photosynthetic prokaryotes, which have various special physiological functions and metabolic abilities. They can use their photosynthesis and metabolic engineering to transform organic and inorganic substances in the environment into harmless or useful substances, which have very wide applications in environmental remediation [150,151]. By modifying cyanobacterial chassis cells, they can degrade and absorb specific pollutants. Here are some examples of using synthetic biology to modify cyanobacterial chassis cells for better environmental remediation.

Touliabah et al. [152] introduced a novel bioremediation method using modified cyanobacteria to degrade various organic pollutants in water. By using genetic engineering, they increased the number of active groups on the algal cell wall, stimulated polysaccharide secretion, and enhanced the effect of algal adsorption of pollutants. Kuritz and Wolk [153] demonstrated the natural ability of two filamentous cyanobacteria to degrade organochlorine pesticides (γ-hexachlorocyclohexane) and enhanced this ability by genetic engineering. They also provided qualitative evidence that these two strains can be genetically engineered to degrade another chlorinated pollutant, 4-chlorobenzoic acid. This method uses the photosynthesis and metabolic engineering of cyanobacteria to transform organic pollutants into harmless or useful substances, providing a new way for environmental protection and resource utilization. Erbe et al. [154] used a shuttle vector to clone the cDNA encoding mouse metallothionein, forming a translational fusion with the bacterial chloramphenicol acetyltransferase gene and expressed it in the cyanobacterium *Synechococcus elongatus* PCC 7942. This method increased the cadmium ion tolerance and accumulation of cyanobacteria by two to five times, providing a new strategy for using cyanobacteria as heavy metal bioindicators and biosorbents.

Compared with other methods of treating pollutants, using modified cyanobacterial chassis cells has many advantages. Cyanobacterial biodegradation can completely or partially degrade pollutants in a short time, reduce or eliminate the content and toxicity of pollutants, and distinguish different types and levels of pollutants, avoiding the impact on nontarget substances. Cyanobacterial biodegradation can adapt to different environmental conditions and can degrade under different temperatures, light, pH, salinity, and other conditions. At the same time, it can regulate the expression and activity of gene circuits, control the speed and degree of degradation, and avoid excessive interference or damage to the environment. Cyanobacterial biodegradation can use light energy and inorganic substances in water for photosynthesis and can also use pollutants as carbon sources or electron acceptors, achieving environmental purification and resource utilization. At the same time, it can produce some useful substances, such as hydrogen, methane, lipids, pigments, etc., achieving environmental restoration and energy recovery, increasing the added value and sustainability of biodegradation.

## 6. Concluding Remarks and Future Perspectives

The synthetic biology of cyanobacteria offers great promise for enhancing the production of high-value products and chemicals in photoautotrophic chassis cells, which is complementary to the modification of synthetic biological effects of cyanobacteria chassis and environmental remediation. However, the application of synthetic biology tools and strategies to the artificial design and optimization of light-driven cyanobacteria cell factories is still in its infancy, and there are still huge challenges for future synthetic biologists, such as improving genetic tool manipulation, discovering and designing efficient cyanobacteria chassis, simplifying and optimizing metabolic regulation, and improving and combining metabolic models.

It is worth noting that cyanobacteria have a positive impact on environmental adaptability after the transformation of cyanobacteria into chassis cells. The growth rate, transformation efficiency, stress resistance, and other frequently studied aspects of cyanobacteria have been significantly improved. Researchers can further expand the application scope of cyanobacteria in various fields and explore the potential uses of cyanobacteria chassis. We believe that with unremitting efforts, synthetic biologists can open a bright future for large-scale applications of green cell factories built from photoautotrophic chassis and applied to various biotechnologies.

## Figures and Tables

**Table 2:** Recent progress in modifying endogenous genes of cyanobacteria by genetic engineering to enhance robustness.

Strain	Strategy	Genes	Objective	References
*Synechocystis* sp. PCC 6803 *Synechococcus elongatus* PCC 7942	Overexpression	The shikimate kinase gene	Improved high light and high temperature tolerances	[28]
*Anabaena* sp. PCC 7120	Overexpression	*groESL*	Improved high salt and high temperature tolerances	[43]
*Synechocystis* sp. PCC 6803	Overexpression	*clpB1*, *dnaK2*	Heat tolerance	[44]
*Synechococcus elongatus* PCC 7942 *Synechocystis* sp. PCC 6803	Overexpression	*hspA*	Improved high light, high temperature, high salt, and oxidative tolerances	[41,45]
*Synechocystis* sp. PCC 6803	Overexpression	*sigB*	Improved high temperature and butanol tolerances	[17]
*Synechocystis* sp. PCC 6803	Overexpression	*sodB*	Improved alcohol tolerance	[46]
*Anabaena* sp. PCC 7120	Overexpression	*all3940*	Improved heavy metals, UV, salts, and temperatures tolerances	[47]
*Anabaena* sp. PCC 7120	Overexpression	*alr2882*	Improved metal tolerance	[48]
*Anabaena* sp. PCC 7120	Overexpression	*all3940*	Improved salinity, heat, heavy metals, pesticide, and nutrient starvation tolerances	[47]
*Anabaena* sp. PCC 7120	Overexpression	*groESL*	Improved heat and salinity tolerances	[43]
*Synechocystis* sp. PCC 6803	Knockout	*slr0724*	Improved n-hexane tolerance	[49]
*Synechococcus elongatus* PCC 7942	Mutation	*atpA*	Improved stress tolerance	[50]

**Table 3:** Maximum titer of four types of high-value products produced by engineering cyanobacteria in recent years.

State	Product	Cyanobacterial Chassis	Titer/Culture Time (Recombinant)	References
Macromolecular substance	PHA	*Synechocystis* sp. PCC 6803	533 mg/L	[77]
	PHB	*Synechococcus elongatus* UTEX 2973	420 mg/L (10 d)	[78]
	PLA	*Synechococcus elongatus* PCC 7942	108 mg/L	[79]
Water insoluble liquid substance	Squalene	*Synechococcus elongatus* UTEX 7942	79.2 mg/g dry cell weight (DCW)	[80]
	α-Farnesene	*Synechococcus* sp. PCC 7002	0.6 mg/L (4 d)	[81]
	Limonene	*Synechococcus elongatus* UTEX 2973	16.4 mg/L (2 d)	[82]
	FFA	*Synechocystis* sp. PCC 11901	1.54 g/L (5 d)	[20]
	Isobutanol	*Synechococcus elongatus* PCC 7942	550 mg/L (8 d)	[83]
	Isoprene	*Synechocystis* sp. PCC 6803	1.26 g/L (21 d)	[84]
	Astaxanthin	*Synechocystis* sp. PCC 6803	29.6 mg/g (DCW)	[85]
Gas	Ethylene	*Synechocystis* sp. PCC 6803	2463 μL (L/h/OD)	[86]
	Hydrogen	*Nostoc punctiforme* ATCC 29133	300 µmol/L/h	[87]
Water Soluble but High-Value Products	Astaxanthin	*Synechocystis* sp. PCC 6803	1.089 µg/mL/OD_730_	[88]
	2,3-butanediol	*Synechococcus elongatus* PCC 7942	12.6 g/L	[89]
	Sucrose	*Synechococcus elongatus* UTEX 2973	8.7 g/L	[90]
	Ethanol	*Synechocystis* sp. PCC 6803	5.5 g/L (26 d)	[14]
	3-hydroxypropionic acid	*Synechocystis* sp. PCC 6803	837.18 mg/L (6 d)	[91]
	L-Lysine	*Synechococcus elongatus* UTEX 2973	556 mg/L (5 d)	[92]
	Glutarate	*Synechococcus elongatus* UTEX 2973	67.5 mg/L (4 d)	[92]
	Cadaverine	*Synechococcus elongatus* UTEX 2973	55.3 mg/L (4 d)	[92]
	Alkaloids	*Synechococcus elongatus* UTEX 2973	0.75–3 mg/L (30 h)	[93]
	Acetone	*Synechococcus elongatus* PCC 7942	0.41 g/L/d	[94]
	1-Butanol	*Synechococcus elongatus* PCC 7942	600 mg/L/d	[95]
	1,3-Propanediol	*Synechococcus elongatus* PCC 7942	0.338 g/L (20 d)	[96]
	Glycerin glucoside (GG)	*Synechocystis* sp. PCC 6803	1.64 g/L (32 d)	[97]
	Succinic acid	*Synechocystis* sp. PCC 6803	1.8 g/L (3 d)	[98]

## Data Availability

No new data were created or analyzed in this study.
